# Pre-eclampsia: Universal Screening or Universal Prevention for Low and Middle-Income Settings?

**DOI:** 10.1055/s-0040-1713803

**Published:** 2021-01-29

**Authors:** Leandro Gustavo De Oliveira, Angélica Lemos Debs Diniz, Caio Antônio de Campos Prado, Edson Vieira Da Cunha Filho, Francisco Lázaro Pereira De Souza, Henri Augusto Korkes, José Geraldo Ramos, Maria Laura Costa, Mário Dias Corrêa Junior, Nelson Sass, Ricardo De Carvalho Cavalli, Sérgio Hofmeister De Almeida Martins-Costa, José Carlos Peraçoli

**Affiliations:** 1 Department of Gynecology and Obstetrics, Botucatu Medical School, Universidade Estadual Paulista “Júlio de Mesquita Filho,” Botucatu, SP, Brazil; 2 Department of Obstetrics and Gynecology, Faculty of Medicine, Universidade Federal de Uberlândia, Uberlândia, MG, Brazil; 3 Department of Gynecology and Obstetrics, Faculty of Medicine, Universidade de São Paulo, Ribeirão Preto, SP, Brazil; 4 Gynecology and Obstetrics Training Center, School of Medicine, Pontifícia Universidade Católica do Rio Grande do Sul, Porto Alegre, RS, Brazil; 5 Department of Tocoginecology, Centro Universitário Lusíada, Santos, SP, Brazil; 6 Department of Obstetrics and Gynecology, Faculty of Medicine, Pontifícia Universidade Católica de São Paulo, Sorocaba, SP, Brazil; 7 Department of Gynecology and Obstetrics, Faculty of Medicine, Universidade Federal do Rio Grande do Sul, Porto Alegre, RS, Brazil; 8 Department of Gynecology and Obstetrics, Faculty of Medical Sciences, Universidade Estadual de Campinas, Campinas, SP, Brazil; 9 Department of Gynecology and Obstetrics, Faculty of Medicine, Universidade Federal de Minas Gerais, Belo Horizonte, MG, Brazil; 10 Escola Paulista de Medicina, Universidade Federal de São Paulo, São Paulo, SP, Brazil

**Keywords:** preeclampsia, screening, prevention, aspirin, calcium, pré-eclâmpsia, rastreamento, prevenção, ácido acetilsalicílico, cálcio

## Abstract

Pre-eclampsia (PE) is a severe disorder that affects up to 8% of all pregnancies and represents an important cause of maternal and perinatal morbidity and mortality. The screening of the disease is a subject of studies, but the complexity and uncertainties regarding its etiology make this objective a difficult task. In addition, the costs related to screening protocols, the heterogeneity of the most affected populations and the lack of highly effective prevention methods reduce the potential of current available algorithms for screening. Thus, the National Specialized Commission of Hypertension in Pregnancy of the Brazilian Association of Gynecology and Obstetrics Federation (Febrasgo, in the Portuguese acronym) (NSC Hypertension in Pregnancy of the Febrasgo) considers that there are no screening algorithms to be implemented in the country to date and advocates that Aspirin and calcium should be widely used.

## Resumo

A Pré-eclâmpsia (PE) é uma doença grave que acomete ∼ 8% das gestações e representa importante causa de morbimortalidade, tanto materna quanto perinatal. O rastreamento da doença é motivo de estudos, porém a complexidade e as incertezas quanto a sua etiologia tornam esse objetivo bastante difícil. Além disso, os custos relacionados com o rastreamento, a heterogeneidade das populações mais afetadas e ainda a falta de métodos de prevenção de grande eficácia reduzem o potencial dos algoritmos de rastreamento. Assim, a Comissão Nacional Especializada sobre Hipertensão na Gravidez da Federação Brasileira das Associações de Ginecologia e Obstetrícia (CNE Hipertensão na Gravidez da FEBRASGO) considera que não há algoritmos de rastreamento que possam ser aplicados no país nesse momento e defende a utilização dos métodos de prevenção como ácido acetilsalicílico e cálcio de maneira ampla.

## Introduction

Pre-eclampsia (PE) is a severe disorder, affecting up to 8% of all pregnancies worldwide. It represents an important cause of maternal and perinatal morbidity and mortality. Most of the adverse outcomes occur in low and middle-income settings. 1

Regarding Brazil, the latest revised and available data on maternal mortality is from 2018. 2 The maternal mortality ratio for that year was 56 deaths per 100,000 live births. In 2008, the documented ratio was 57 per 100,000 live births, with clear stagnation over 10 years, and with PE accounting for 20% of these deaths. The impact of PE on perinatal outcomes is not correctly estimated, but it is known that 18% of all preterm births in Brazil are related to PE and that ∼ 55% of these cases are elective preterm births due to maternal indications related to PE. 3 4 It is possible that many of these indications are iatrogenic due to the lack of a reliable model of risk stratification to be used in clinical practice.

Considering the importance of PE worldwide and mainly in Brazil, screening pregnant women at higher risk for developing the disease is essential. However, the prediction of PE is a difficult task, because of its complex etiology, gaps in its pathophysiology, diversity of clinical presentations and heterogeneity among populations.

Recently, the International Federation on Gynecology and Obstetrics (FIGO) issued its position on universal screening for PE. 5 However, this recommendation is still controversial and the National Specialized Commission of Hypertension in Pregnancy of the Brazilian Association of Gynecology and Obstetrics Federation – (Febrasgo, in the Portuguese acronym) (NSC Hypertension in Pregnancy of the Febrasgo) considers the importance of also presenting its position, to reinforce the local recommendations on PE.

## FIGO Recommendations

A recent publication by FIGO recommends universal screening for PE in the 1 ^st^ trimester of pregnancy using an algorithm that involves maternal characteristics, evaluation of mean arterial blood pressure, uterine artery Doppler and serum levels of Placental Growth Factor (P1GF) and Pregnancy Associated Pregnancy Protein - A (PAPP-A). This recommendation was based primarily on the results of the following studies that screened pregnant women in the first trimester (11–13 weeks):

1) Screening of 58,884 singleton pregnant women. Of these women, 1,426 (2.4%) developed PE. The detection rate was 77% for preterm PE (< 37 weeks) and 54% for total PE, with a 10% false-positive rate. 6
2) Screening of 35,948 singleton pregnant women. Of these women, 1,058 (2.9%) developed PE. The detection rates for preterm and term PE were 75% and 47%, respectively, with a 10% false-positive rate. 7
3) Screening of 26,941 singleton pregnant women in 13 maternity hospitals in 6 different countries (United Kingdom, Spain, Italy, Belgium, Greece and Israel) - ASPRE study. The detection rates for preterm and term PE were 77% and 43%, respectively, with a false-positive rate of 9.2%. 8 This study identified 2,971 women at risk for developing preterm PE. Of these pregnant women considered to be at higher risk, 1,776 were randomized and 798 received 150mg of Aspirin for prevention of PE and 822 received placebo. At the end of the study, 13 women developed PE in the study group versus 35 in the placebo group.

To prevent PE, the FIGO recommends the use of Aspirin at a dose of 150mg orally at night and calcium supplementation for women with inadequate calcium intake (< 800mg/day), emphasizing that this prevention should be based on positive screening.

Despite the recommendation based on positive screening, the FIGO recognizes the following risk factors to develop PE: maternal age ≥ 35 years old, nulliparity, previous history of PE, pregnancy interval < 12 months or > 72 months, assisted reproduction, family history of PE (mother and sisters who had PE), obesity (body mass index [BMI] ≥ 30), Afro-Caribbean race and presence of clinical conditions (chronic arterial hypertension, diabetes 1 and 2, kidney disease, systemic lupus erythematosus and antiphospholipid antibody syndrome). Finally, the FIGO recognizes that areas where the costs of the complete screening for PE are not affordable could implement an alternative screening based on clinical risk factors and assessment of mean arterial blood pressure in the 1 ^st^ trimester.

### Recommendations of the NSC Hypertension in Pregnancy of the Febrasgo

The NSC-Hypertension Brazil is comprised of a group of specialists dedicated to the study and assistance of pregnant women diagnosed with PE in the country. These professionals represent important Universities in Brazil and are responsible for developing guidelines that can be followed at a national level. 9 10 The NSC Hypertension in Pregnancy of the Febrasgo met to discuss the FIGO recommendations regarding universal screening for PE in the 1 ^st^ trimester, considering the available evidences, as well as the economic impact of such recommendation for a low-income country such as Brazil. After this discussion, the following considerations were established:

Regarding the use of algorithms involving clinical characteristics, biophysical and biochemical markers, it is possible to note a series of studies suggesting that such screening model is not universally accepted. Townsend et al recently evaluated the main reviews on this issue in the medical literature. 11 The authors pointed out a great heterogeneity among the markers used to predict PE and only half of the available reviews assessed the quality of the studies included. Despite lack of quality assessment, some predictors presented better performances. Body mass index > 35 kg/m2 presented specificity of 92% (95% confidence interval [CI], 89–95%) and sensitivity of 21% (95% CI, 12–31%); BMI > 25 kg/m2 had specificity of 73% (95% CI, 64–83%) and sensitivity of 47% (95% CI, 33–61%); first-trimester uterine Doppler with pulsatility or resistance index > 90 ^th^ had specificity of 93% (95% CI, 90–96%) and a sensitivity of 26% (95%CI, 23–31%) and PlGF concentration presented specificity of 89% (95% CI, 89–89%) and sensitivity of 65% (95% CI, 63–67%). This review of the reviews concluded that no single marker can be used in the clinical practice, but the combination of different markers appears to be promising. However, none of the proposed screening models has undergone external validations. Di Martino et al evaluated the performance of the algorithm suggested by the group of researchers related to the recommendations published by the FIGO. 12 After applying this algorithm to 11,632 Italian pregnant women, the authors demonstrated a detection rate of 58.2% for early PE (< 34 weeks) and 41.8% for late PE (≥ 34 weeks). The studies cited in the FIGO's recommendation presented detection rates of no more than 77% for early PE and were clearly unable to identify women who later developed late or term PE. 6 7 8

It is important to consider the weight of each marker proposed in screening models, costs and logistics required to incorporate each one in places with restricted structural conditions or few financial resources. When considering the weight of each marker, O'Gorman et al demonstrated that maternal characteristics had a detection rate of 53% (40–65%) for PE before 32 weeks and that the addition of mean blood pressure assessment raised this rate to 65% (52–76%). 7 Bearing in mind the weight of each marker used for PE screening leads us to question whether there is sufficient benefit to invest financial resources in an attempt to raise detection rates that would go from 65% to 77%. So far, the NSC Hypertension in Pregnancy of the Febrasgo does not recognize this benefit. In practical terms, the ASPRE study initially screened 26,941 pregnant women and, among these women, the total number that would develop preterm PE whether no intervention had been implemented would be ∼ 70 women (0,26%). 8 Once again, the NSC Hypertension in Pregnancy of the Febrasgo does not consider the existence of a balance among benefits of detection, costs and logistics required to recommend universal screening based on the suggested algorithm, mainly in the Brazilian regions where there are no financial resources.

Another question that deserves an answer at this point is whether there is any contraindication in recommending Aspirin and calcium for more pregnant women than those screened by expensive algorithms. Recent studies indicate that the benefits of using low-dose Aspirin go beyond the prevention of PE. Andrikopulou et al demonstrated that the use of Aspirin in nulliparous women without morbidities led to important reduction in preterm births before 34 weeks of gestation. 13 A recent trial (ASPIRIN) demonstrated that the introduction of Aspirin between 6 and 13 weeks plus 6 days for nulliparous women led to considerable reduction in preterm births and perinatal mortality. 14

Mallampati et al studied the outcomes related to the universal use of Aspirin, without clinical or laboratory screening. 15 The authors demonstrated a significant reduction in the incidence of PE, mainly preterm PE, from 311 to 148 cases per 1,000 pregnancies. There was also a significant reduction in costs when the universal prescription was compared with the screening by biomarkers and ultrasound (∼ US$192.16 per patient). Additionally, the implementation of biomarkers and ultrasound for PE screening increased costs by US$19,216,251 per 100,000 pregnant women. These results still need better analyses, mainly regarding adverse events or side effects, but they are initially very interesting.

Calcium supplementation for women with low calcium intake has been recognized by the World Health Organization (WHO). 16 In Brazil, calcium intake by women at childbearing age is ∼ 500mg/day, that is, below current recommendations. 17 Sun et al demonstrated in a systematic review that the supplementation with low doses of calcium (from 600mg/day) significantly reduced the incidence of PE ( *p* < 0,001), even in low-risk populations (risk ratio [RR]: 0.32; 95%CI: 0.18–0.59) and mainly in developing countries (RR: 0.41; 95%CI: 0.29–0.58). 18 Therefore, the NSC Hypertension in Pregnancy of the Febrasgo understands that recommending Aspirin and calcium supplementation for pregnant women with clinical risk factors for PE and all nulliparous women, especially those in the public health system in Brazil, can be an important strategy to improve maternal and perinatal outcomes.

Regarding the recommendation to use Aspirin at a dose of 150mg/day, we emphasize that the ASPRE study did not compare different doses and received criticisms in this regard. 8 19 20 Brazil has 100mg tablets that are supplied by the public health system. This concentration is already higher than that recommended in North America, which is 85mg/day. Thus, the NSC Hypertension in Pregnancy of the Febrasgo does not find subsidies to recommend an increase in the Aspirin doses at the moment and since the country does not have different formulations available in the public health system, the NSC Hypertension in Pregnancy of the Febrasgo maintains the recommendation of Aspirin at 100mg/day. The drug should be started at 12 weeks of gestation, and some authors claim that the benefits are achieved with its introduction up to 16 weeks of gestation. However, a recent meta-analysis confirms that the benefits are consistent whether the medication is commenced up to 20 weeks of pregnancy. 21 Therefore, this is the recommendation from the NSC Hypertension in Pregnancy of the Febrasgo.

### Final Considerations

The NSC Hypertension in Pregnancy of the Febrasgo emphasizes the importance of maintaining the investigation of effective methods for screening PE, mainly according to the characteristics of each population. The various phenotypes of PE suggest that different populations may present different risk factors and such interpretation needs to be considered. The FIGO itself, in its publication, recognizes that the screening methods must be adequate for each setting and that the existing resources must be respected. Thus, the NSC Hypertension in Pregnancy of the Febrasgo maintains, at this time, the recommendation for screening PE based on clinical and epidemiological characteristics. Finally, the NSC Hypertension in Pregnancy of the Febrasgo reinforces its position that the main impact in reducing morbidity and mortality due to PE, in our scenario, is to improve the quality of antenatal care, risk identification and timely diagnosis, to ensure proper management to the different clinical presentations of PE.
